# The influence of pterygium on corneal densitometry evaluated using the Oculus Pentacam system

**DOI:** 10.3389/fmed.2023.1184318

**Published:** 2023-06-15

**Authors:** Jing Zhang, Liyun Zhang, Huiling Hu, Liangnan Sun, Wenling He, Zhe Zhang, Jiantao Wang, Danyao Nie, Xinhua Liu

**Affiliations:** ^1^Shenzhen Eye Hospital, Jinan University, Shenzhen Eye Institute, Shenzhen, Guangdong, China; ^2^Shenzhen Key Laboratory of Ophthalmology, Shenzhen Eye Hospital, Postgraduate Training Base of Jinzhou Medical University, Shenzhen, Guangdong, China

**Keywords:** corneal densitometry, pterygium, pterygium surgery, Pentacam, corneal topography

## Abstract

**Purpose:**

To assess the effect of pterygium on corneal densitometry (CD) values.

**Methods:**

One hundred and nine patients (155 eyes) with primary pterygium were divided into a severe pterygium group (79 eyes) and a mild-to-moderate pterygium group (76 eyes) according to pterygium severity. Among them, 63 patients had monocular pterygium; and 25 patients (38 eyes) underwent pterygium excision combined with conjunctival autograft follow-up. A Pentacam anterior segment analyzer was used to obtain the CD values and corneal morphological parameters, including central corneal thickness (CCT), flat-axis keratometry (K1), steep-axis keratometry (K2), corneal astigmatism, irregular astigmatism, and spherical aberration. CD was subdivided into four concentric radial regions based on corneal diameter and three layers according to depth.

**Results:**

CD values at 0–12 mm of the anterior 120 μm layer, 0–10 mm of the center layer and full thickness, and 2–6 mm of the posterior 60 μm layer were significantly higher in eyes affected by pterygium than in the contralateral unaffected eyes (all *P* < 0.05). CD values were significantly higher in the severe pterygium group than in the mild to moderate pterygium group (all *P* < 0.05). Corneal astigmatism, irregular astigmatism, K1, K2, CCT, and spherical aberration correlated with CD values in eyes with pterygium (all *P* < 0.05). CD values at 6–10, 0–12 mm in the anterior 120 μm layer and full thickness, 10–12 and 0–12 mm in the center layer were significantly decreased 1 month after pterygium surgery compared with those before surgery (all *P* < 0.05).

**Conclusion:**

Patients with pterygium had increased CD values, particularly in the anterior and central layers. CD values were correlated with pterygium severity grading and corneal parameters. Pterygium surgery partially reduced the CD values.

## Introduction

Pterygium is a common ocular surface disease with an overall global incidence as high as 12% (1). Pterygium is characterized by degeneration, hypertrophy, and hyperplasia of the bulbar conjunctiva and the subconjunctival tissue of the palpebral fissure. It progresses into the cornea and can destroy Bowman’s membrane and superficial stromal layer of the cornea (2). The clinical hazards of pterygium include affecting aesthetics and corneal morphology, increased wavefront aberrations, irregular astigmatism of the cornea and decreased visual quality. In severe cases, decreased visual acuity and limited eye movements may also occur (3–7). Currently, no specific medical treatments are available. Pterygium excision combined with conjunctival autograft transplantation is currently the mainstream surgical approach owing to its low recurrence rate and few complications (8–10).

Previous studies have found that corneal densitometry (CD) measured by Pentacam gradually increases with age in healthy subjects (11–14). In addition to Pentacam, anterior segment optical coherence tomography and corneal confocal microscopy have also been reported to be useful for measuring CD values (15, 16). However, only Pentacam has been widely used in the study of different corneal diseases and surgeries due to its advantages of easy measurement, automatic, non-contact. Many ocular surface diseases, such as keratoconus, vernal keratitis, dry eye, and primary congenital glaucoma have an impact on CD (17–20). Anterior segment surgeries including small incision lenticule extraction (SMILE), femtosecond Laser-assisted *In Situ* Keratomileusis (FS-LASIK), implantable collamer lens (ICL) V4c implantation, and intrastromal Ferrara ring implantation also affect CD (21–23).

Numerous studies have investigated and documented the impact of different pterygium sizes and pterygium surgeries on corneal status, including astigmatism, keratometry, and corneal topography (3, 4, 24, 25). The evaluation of pterygium-induced CD changes and the impact of pterygium surgery on CD using Scheimpflug imaging has not been documented in the literature. Therefore, this study aimed to investigate pterygium-induced CD changes and the effects of pterygium surgery on CD.

## Patients and methods

### Patients

This was a retrospective study. Patients with pterygium who visited Shenzhen Eye Hospital between January 2019 and March 2022 were included in this retrospective study. The study complied with the Declaration of Helsinki and the study protocol was approved by the Ethics Committee of Shenzhen Eye Hospital (No.2022KYPJ010). The requirement for individual consent was waived. The inclusion criteria were age >18 years and primary pterygium tissue growth in the nasal palpebral fissure area. Exclusion criteria were recurrent pterygium, temporal pterygium, pseudopterygium, conjunctival cyst, keratoconjunctival tumor, history of ocular trauma, history of ophthalmic surgery or other ocular diseases affecting CD, and history of systemic diseases, such as hyperthyroidism, autoimmune diseases, and tumors.

Pterygium severity was graded according to the extent of pterygium growth, vascularization, and thickness according to previous studies (26). Severity was graded as mild (3–5 points), moderate (6–8 points), or severe (9–11 points). One hundred and fifty-five eyes of 109 patients with primary pterygium were included in this study. According to the severity of pterygium, three eyes graded as mild, 73 eyes as moderate, and 79 eyes as severe. As there were fewer patients with mild pterygium, they were combined with patients with moderate pterygium. As a result, only the mild-to-moderate group was compared with the severe group.

### Examinations

All subjects underwent routine ophthalmic examinations, including visual acuity, slit-lamp microscopy, intraocular pressure, fundus examination, and anterior segment photography. A Pentacam three-dimensional anterior segment analyzer (Oculus Optikgerate GmbH, Wetzlar, Germany) was used by the same experienced doctor to obtain corneal morphological parameters and CD values. This examination was performed in a darkroom with the subject seated and the lower jaw placed on rest with fixation of the target in both eyes. Twenty-five Scheimpflug images of the cornea with different meridians were automatically acquired using three-dimensional scanning mode. CD, cornea-related parameters including central corneal thickness (CCT), flat-axis keratometry (K1), steep-axis keratometry (K2), corneal astigmatism, irregular astigmatism, and spherical aberration, can all be obtained by Pentacam measurements. CD ranges from 0 to 100 gray-scale units (GSU), with 0 representing complete clearing and 100 representing complete opacification of the cornea. CD was subdivided into four concentric radial regions based on corneal diameter, including the corneal apex to a diameter of 2, 2–6, 6–10, and 10–12 mm. The cornea was divided into three layers according to depth: the anterior 120 μm layer, center, and posterior 60 μm for the evaluation of CD.

### Surgical methods

Thirty-eight eyes in 25 patients underwent pterygium excision combined with conjunctival autograft surgery. All surgeries were performed by a single experienced surgeon (JZ). Subconjunctival infiltration anesthesia was administered using 2% lidocaine. The bulbar conjunctiva of the pterygium neck was cut approximately 2 mm posterior to the limbus, and the subconjunctival tissue was exposed and bluntly separated. The pterygium tissue between the conjunctiva and sclera was bluntly separated. The pterygium roots were cut before the semi-lunar fold. The head of the pterygia on the cornea was removed via blunt dissection. The extent of the conjunctival defects was measured using calipers. Free conjunctival grafts were obtained from the superior temporal conjunctiva and fixed in the nasal conjunctival defect area using interrupted sutures (10-0 nonabsorbable silk). Postoperative treatment included tobramycin dexamethasone ophthalmic solution (s.a. ALCON-COUVREUR n.v.) four times daily for 1 month. Tobramycin dexamethasone eye ointment (s.a. ALCON-COUVREUR n.v.) was instilled every night for 14 days and ofloxacin eye drops (Santen Pharmaceutical Co., Ltd.) four times daily for 7 days. All patients underwent routine examinations including slit-lamp microscopy, visual acuity and intraocular pressure before surgery and at 1, 7, and 30 days after surgery. Conjunctival suture removal was performed one week after surgery. A Pentacam anterior segment analyzer was performed preoperatively and at 1 month after surgery.

### Statistical analysis

Statistical analyses were performed using the IBM SPSS Statistics version 21 (IBM Corp., Armonk, NY, USA). Normality tests were performed using the Kolmogorov–Smirnov test. The statistical description of parameters conforming to the normal distribution is expressed as the mean ± SD, and that of parameters not conforming to the normal distribution is expressed as P50 (P25-P75). The Mann-Whitney U test was used to compare two independent samples of data that did not conform to a normal distribution. The chi-squared test was used to compare two categorical variables. Pairwise comparisons were performed using a paired Wilcoxon test. Correlation coefficients were assessed using Spearman correlation analysis. Statistical significance was set at *P* < 0.05.

## Results

There are three different grouping methods to compare corneal topography parameters and CD values in patients with pterygium. First, the comparison between pterygium eyes and self-control eyes in patients with monocular pterygium. Second, comparisons were made according to pterygium severity. Finally, pterygium was compared preoperatively and 1 month postoperatively. Demographic characteristics of pterygium patients in three different grouping methods are shown in Table 1.

**TABLE 1:** Demographic characteristics of pterygium patients with three different grouping methods.

	Grouping method		
	Monocular Patients	Severity	Surgery
*N* (eyes)	63 (63)	109 (155)	25 (38)
Age, y Mean ± SD (range)	60.11 ± 13.57 (31, 87)	62.50 ± 12.35 (31, 87)	69.36 ± 10.73 (33, 85)
Sex, eyes Male/female	28/35	63/92	9/16
Eye Monocular/binocular	63/0	89/33	13/12
Grade, eyes Mild/moderate/severe	2/30/31	3/73/79	0/14/24

### Comparison between pterygium eyes and self-control eyes

**FIGURE 1:**
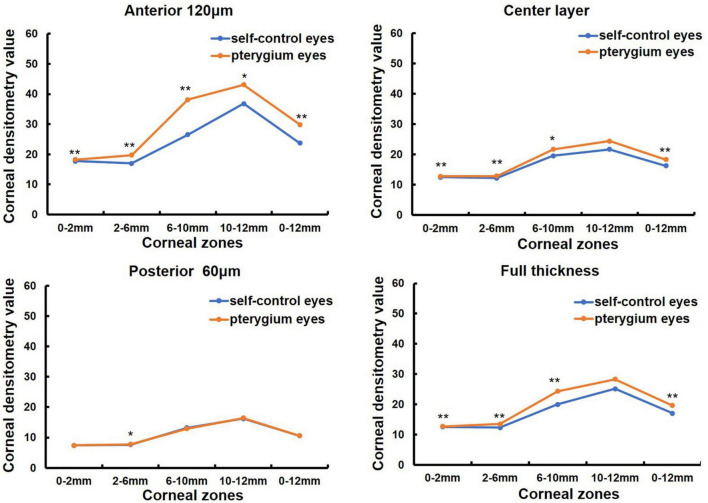
Comparison of corneal densitometry (CD) value at different corneal zones and corneal depths between the pterygium eye and the contralateral eye in 63 monocular pterygium patients, *P < 0.05,**P < 0.001.

Sixty-three patients with monocular pterygium underwent examination of the pterygium eye and contralateral eye (in Table 1). There were 28 males and 35 females, with a mean age of 60.11 ± 13.57 years (range: 31–87 years). K1 and spherical aberrations were significantly lower in pterygium eyes than in contralateral eyes (all *P* < 0.05, Table 2). K2, corneal astigmatism and irregular astigmatism were significantly higher in the eyes with pterygium than in the contralateral eye (all *P* < 0.05, Table 2). There were no significant differences in CCT between the pterygium and contralateral eyes (*P* > 0.05, Table 2). CD values were significantly increased in pterygium eyes compared with contralateral eyes at 0–2, 2–6, 6–10, 0–12 mm in the anterior 120 μm, center, and full thickness, 10–12 mm in the anterior 120 μm, and 2–6 mm in the posterior 60 μm (Z = −4.157, *P* = 0.000; Z = −6.081, *P* = 0.000; Z = −6.213, *P* = 0.000; Z = −6.555, *P* = 0.000; Z = −4.359, *P* = 0.000; Z = −5.506, *P* = 0.000; Z = −3.387, *P* = 0.001; Z = −4.684, *P* = 0.000; Z = −3.577, *P* = 0.000; Z = −5.615, *P* = 0.000; Z = −4.982, *P* = 0.000; Z = −5.949, *P* = 0.000; Z = −2.444, *P* = 0.015; Z = −2.857, *P* = 0.004). However, there was no significant difference between the eyes with pterygium and the contralateral eyes in the center and full thickness of 10–12 mm and, posterior 60 μm of 0–2, 6–10, 10–12, and 0–12 mm (all *P* > 0.05, Figure 1).

**TABLE 2:** Comparison of corneal topographic parameters between pterygium eyes and contralateral eyes in 63 patients.

Groups	K1 (D)	K2 (D)	Astigmatism (D)	CCT (μ m)	Spherical aberration	Irregular astigmatism (D)
Pterygium eye	41.40 (38.90, 43.50)	44.70 (43.30, 46.30)	1.90 (0.70, 6.90)	550.00 (522.00, 572.00)	0.25 (0.12, 0.41)	0.50 (0.28, 1.52)
Contralateral eye	43.60 (42.70, 45.00)	44.40 (43.40, 45.80)	0.80 (0.40, 1.10)	540.00 (520.00, 558.00)	0.37 (0.23, 0.48)	0.17 (0.12, 0.25)
Z	−5.474	−2.075	−5.330	−1.798	−3.310	−6.381
*P*	0.000**	0.038*	0.000**	0.072	0.001**	0.000**

K1, flat-axis keratometry; K2, steep-axis keratometry; CCT, central corneal thickness; non-normally distributed parameter values are expressed as P50 (P25-P75).

All comparison made using paired Wilcoxon test.

*Statistical significance, *p* < 0.05.

**Statistical significance, *p* < 0.01.

### Comparison of the CD values according to the pterygium severity

**FIGURE 2:**
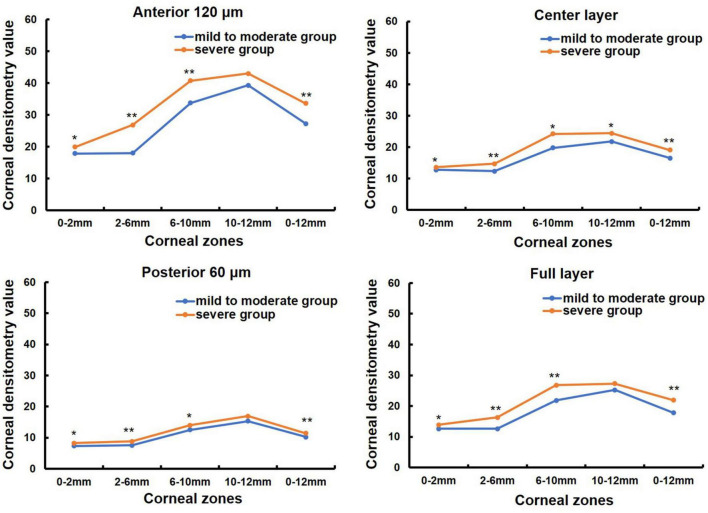
Comparison of corneal densitometry (CD) value of different severity pterygium in different corneal zones and corneal depths, *P < 0.05, **P < 0.001.

One hundred and nine patients (155 eyes) with primary pterygium were divided into mild to moderate (76 eyes) and severe groups (79 eyes) according to pterygium severity (in Table 1). The mean age was 60.14 ± 13.47 (31, 84) years in the mild to moderate group and 64.76 ± 10.77 (33, 87) years in the severe group, and the latter was older than the former (Z = −2.077, *P* = 0.038). There were 33 males and 43 females in the mild to moderate group and 30 males and 49 females in the severe group, with no significant difference between the two groups (χ2 = 1.821, *P* = 0.402). K1 was significantly lower in the severe pterygium group than in the mild and moderate pterygium groups, whereas K2, corneal astigmatism and irregular astigmatism were significantly higher in the severe group than in the mild and moderate groups (all *P* < 0.05, Table 3). CCT and spherical aberration were not significantly different between the two groups (all *P* > 0.05, Table 3). As shown in Figure 2, the CD values of 0–2, 2–6, 6–10, and 0–12 mm in the anterior 120 μm layer, the center layer, the posterior 60 μm layer, and full thickness and 10–12 mm in the center layer were significantly higher in the severe group than in the mild to moderate group (Z = −3.021, *P* = 0.003; Z = −6.189, *P* = 0.000; Z = −4.190, *P* = 0.000; Z = −5.527, *P* = 0.000; Z = −2.904, *P* = 0.004; Z = −4.503, *P* = 0.000; Z = −3.334, *P* = 0.001; Z = −4.378, *P* = 0.000; Z = −3.020, *P* = 0.003; Z = −4.130, *P* = 0.000; Z = −2.733, *P* = 0.006; Z = −3.655, *P* = 0.000; Z = −3.206, *P* = 0.001; Z = −5.686, *P* = 0.000; Z = −3.755, *P* = 0.000; Z = −5.051, *P* = 0.000; Z = −2.535, *P* = 0.011). However, there was no significant difference in CD values of 10–12 mm in the anterior 120 μm, posterior 60 μm, and full-thickness between the two groups (all *P* > 0.05).

**TABLE 3:** Comparison of corneal topographic parameters between mild to moderate group (76 eyes) and severe group (79 eyes).

Groups	K1 (D)	K2 (D)	Astigmatism (D)	CCT (μ m)	Spherical aberration	Irregular astigmatism (D)
Severe	40.20 (35.63, 42.78)	44.85 (43.93, 46.10)	5.40 (1.85, 10.58)	536.50 (511.50, 578.00)	0.28 (0.12, 0.45)	1.04 (0.48, 1.65)
Mild to moderate	42.85 (41.30, 43.80)	44.20 (43.00, 44.70)	1.00 (0.53, 1.88)	540.00 (507.25, 563.00)	0.23 (0.14, 0.37)	0.30 (0.17, 0.54)
Z	−4.767	−3.833	−7.108	−1.188	−0.870	−6.856
*P*	0.000**	0.000**	0.000**	0.235	0.384	0.000**

K1, flat-axis keratometry; K2, steep-axis keratometry; CCT, central corneal thickness; non-normally distributed parameter values are expressed as P50 (P25-P75).

All comparison made using Mann-Whitney U test.

**Statistical significance, *p* < 0.01.

### Correlation between CD and tomographic parameters in patients with pterygium

K1, K2, corneal astigmatism, irregular astigmatism, and pterygium severity grading were significantly correlated with CD values obtained from all layers, including the anterior 120 μm, center, posterior 60 μm, and full thickness in the 0–2 and 2–6 mm corneal zones (all *P* < 0.05, Table 4). CCT and spherical aberration were significantly correlated with the CD values of the anterior 120 μm, center, and full thickness 0–2 and 2–6 mm corneal zones (all *P* < 0.05, Table 4). The CD values of the posterior 60 μm at 0–2 mm were not correlated with spherical aberration and the CD values of the posterior 60 μm at 2–6 mm were not correlated with CCT (all *P* > 0.05, Table 4).

**TABLE 4:** Correlations between corneal densitometry (CD) values and corneal parameters in patients with pterygium (*n* = 155).

Tomographic parameters	Cornea layer	0–2 mm		2–6 mm	
		ρ	*P*	ρ	*P*
K1	Anterior 120 μm	−0.349	0.000**	−0.58	0.000**
	Center layer	−0.241	0.003**	−0.361	0.000**
	Posterior 60 μm	−0.207	0.01*	−0.27	0.001
	Full length	−0.321	0.000**	−0.502	0.000**
K2	Anterior 120 μm	0.226	0.005**	0.34	0.000**
	Center layer	0.19	0.018*	0.322	0.000**
	Posterior 60 μm	0.161	0.046*	0.249	0.002
	Full length	0.237	0.003**	0.348	0.000**
Corneal astigmatism	Anterior 120 μm	0.461	0.000**	0.735	0.000**
	Center layer	0.362	0.000**	0.519	0.000**
	Posterior 60 μm	0.334	0.000**	0.421	0.000**
	Full length	0.451	0.000**	0.668	0.000**
CCT	Anterior 120 μm	0.345	0.000**	0.367	0.000**
	Center layer	0.181	0.024*	0.232	0.004**
	Posterior 60 μm	0.192	0.016*	0.148	0.067
	Full length	0.309	0.000**	0.327	0.000**
Spherical aberration	Anterior 120 μm	0.249	0.002**	0.311	0.000**
	Center layer	0.203	0.011**	0.324	0.000**
	Posterior 60 μm	0.142	0.078	0.26	0.001**
	Full length	0.254	0.001**	0.33	0.000**
Irregular astigmatism	Anterior 120 μm	0.493	0.000**	0.832	0.000**
	Center layer	0.451	0.000**	0.661	0.000**
	Posterior 60 μm	0.409	0.000**	0.553	0.000**
	Full length	0.5	0.000**	0.778	0.000**
Pterygium severity grading	Anterior 120 μm	0.29	0.000**	0.561	0.000**
	Center layer	0.257	0.001**	0.387	0.000**
	Posterior 60 μm	0.252	0.002**	0.335	0.000**
	Full length	0.299	0.000**	0.509	0.000**

K1, flat-axis keratometry; K2, steep-axis keratometry; CCT, central corneal thickness; Correlation coefficients were assessed using a Spearman correlation analysis.

*Statistical significance, *p* < 0.05.

**Statistical significance, *p* < 0.01.

### Comparison before and after pterygium surgery

**FIGURE 3:**
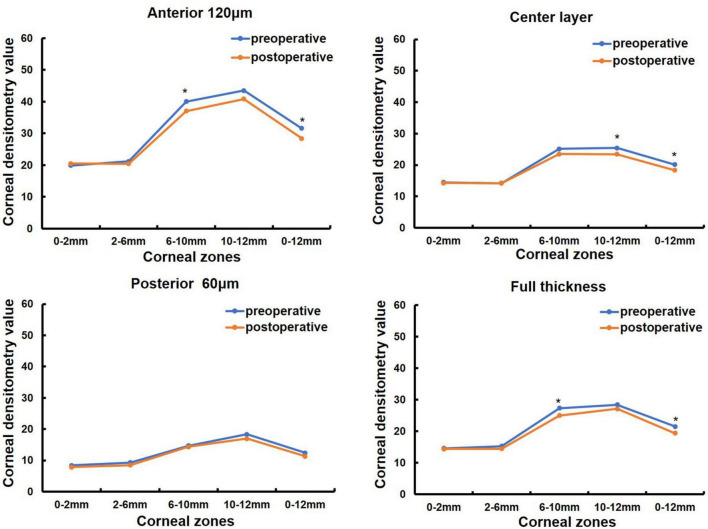
Comparison of corneal densitometry (CD) value at different corneal zones and corneal depths in 38 eyes of 25 patients preoperative and 1 month postoperative, *P < 0.05.

Corneal morphology and CD values were measured before and one month after pterygium surgery in 38 eyes of 25 patients (in Table 1). Among them, 13 patients were monocular and 12 patients were binocular, 9 males and 16 females, with a mean age of 69.36 ± 10.73 (33, 85) years (in Table 1). K1 was significantly increased after surgery compared to that before surgery (*P* < 0.05, Table 5), whereas CCT, corneal astigmatism, and irregular astigmatism values were significantly lower than those before surgery (all *P* < 0.05, Table 5). K2 and spherical aberration were not significantly different before and after surgery (all *P* > 0.05, Table 5). CD values of the anterior 120 μm and full cornea thickness at 6–10 and 0–12 mm, the center layer at 10–12 and 0–12 mm after pterygium surgery were significantly lower than those before surgery (Z = −2.677, *P* = 0.007; Z = −3.205, *P* = 0.001; Z = −2.046, *P* = 0.041, Z = −2.768, *P* = 0.006; Z = −2.016, *P* = 0.044, Z = −2.350, *P* = 0.019, in Figure 3), while CD values of the anterior 120 μm and full thickness of the cornea at 0–2, 2–6, 10–12 mm, center layer at 0–2, 2–6, 6–10 mm and posterior 60 μm layer at 0–2, 2–6, 6–10, 10–12 and 0–12 mm were not significantly different before and after surgery (all *P* > 0.05, Figure 3).

**TABLE 5:** Comparison of corneal topographic parameters before and after pterygium excision combined with conjunctival autograft transplantation surgery 1 month in 38 eyes of 25 patients.

Groups	K1 (D)	K2 (D)	Astigmatism (D)	CCT (μ m)	Spherical aberration	Irregular astigmatism (D)
Preoperative	41.70 (38.75, 43.32)	44.30 (43.23, 45.00)	1.90 (0.63, 4.95)	534.00 (511.25, 569.00)	0.32 (0.18, 0.45)	0.46 (0.26, 0.99)
Postoperative 1M	43.50 (42.50, 44.38)	44.30 (43.33, 45.40)	0.85 (0.50, 1.30)	529.00 (513.25, 559.50)	0.31 (0.16, 0.42)	0.35 (0.20, 0.66)
Z	−4.253	−1.081	−3.897	−2.910	−0.456	−3.416
*P*	0.000**	0.280	0.000**	0.004**	0.648	0.001**

M, month; K1, flat-axis keratometry; K2, steep-axis keratometry; CCT, central corneal thickness; non-normally distributed parameter values are expressed as P50 (P25-P75).

All comparison made using paired Wilcoxon test.

**Statistical significance, *p* < 0.01.

## Discussion

The CD is an important parameter used to assess corneal transparency and health. Our previous study showed that healthy corneal CD values were positively correlated with age and slightly positively correlated with keratometry and spherical aberration (14). Another study reported that CD values increased with age but did not correlate with corneal keratometry and refractive parameters in healthy participants (13). CD values of 0–6 mm in the anterior layer of the keratoconus cornea was reported significantly correlated with maximum keratometry, thinnest corneal thickness and keratoconus severity (18). This study showed that corneal keratometry, spherical aberration, corneal astigmatism, irregular astigmatism, CCT, and pterygium severity correlated with CD values of 0–6 mm in patients with pterygium.

To compare the CD values, normal eyes of patients with monocular pterygium were used as the control group, and confounding factors, such as age, sex, and general condition, were excluded to determine the effect of pterygium on CD. Our results showed that CD values of 0–10 mm in the anterior 120 μm, the center and full thickness, and 2–6 mm in the posterior 60 μm layer were significantly increased in the pterygium affected eyes compared with the contralateral unaffected eyes. CD values of 0–10 mm in the anterior 120 μm, center, and posterior 60 μm and total thickness were higher in patients with severe pterygium than in those with mild or moderate pterygium. Severe pterygium represents more active inflammation and more extensive invasion into the cornea than less severe pterygium. Previous studies have used confocal microscopy to observe the presence of inflammatory cells between the cornea and pterygium, and demonstrated that the density of inflammatory cells was significantly correlated with pterygium mobility score (27). *In vivo* laser scanning confocal microscopy also revealed that the density of corneal basal epithelial cells, anterior stromal cells, endothelial cells, and the length of corneal nerve fibers in the central cornea were higher than in the area near the head of the pterygium. However, dendritic cell density was lower in the former than in the latter (28). Decreased corneal transparency may be due to the irregular arrangement of collagen fibers, uneven distribution of keratocytes, and disruption of the extracellular matrix structure. Pterygium is characterized by cell proliferation and conjunctival tissue growth in the cornea, and inflammatory processes, fibrosis, angiogenesis, and destruction of the extracellular matrix may explain the increased CD observed in patients with pterygium (2). The pterygium severity grading was significantly associated with CD values of 0–2 and 2–6 mm annuli in the anterior 120 μm, central, and full-thickness of patients with pterygium. These findings suggest that CD may be considered as a potential parameter indicator of pterygium severity.

Increases in CD values, measured using the Scheimpflug imaging system, have also been reported in other ocular surface diseases, such as keratoconus, vernal keratoconjunctivitis and dry eye, which may impair corneal transparency (17, 18, 20). CD is negatively correlated with visual acuity in patients with primary congenital glaucoma (PCG) (19). Moreover, the CD value can also be used as a parameter to follow corneal and anterior segment surgical recovery. CD value measured by Scheimpflug imaging decreased in patients with myopia or myopic astigmatism 5 years after small incision lenticule extraction or femtosecond laser-assisted LASIK (21). The author thought that the changes in CD may impact the subjective quality of vision and may be related to symptoms such as glare and halos. Other studies reported that the increase in CD values one day after ICL surgery and cataract surgery may be related to limbal corneal incision edema (23, 29). The increased of CD values at 4 years after ICL surgery correlated with age, preoperative spherical equivalent, uncorrected distance visual acuity, and intraocular pressure (IOP), may be related to the changes in metabolism and collagen fiber arrangement of the corneal endothelial cells due to age (23). CD in the anterior layer of the cornea is reduced after intrastromal corneal ring segments implantation in patients with keratoconus and is correlated with corneal keratometry (22).

The results of this study showed that K1 was significantly increased, and CCT, corneal astigmatism, and irregular astigmatism values were significantly decreased after pterygium excision combined with conjunctival autograft transplantation. Our findings are consistent with those of other studies (30). After pterygium excision, the anterior cornea became significantly steeper, CCT became thinner, and the total astigmatism, anterior corneal astigmatism asymmetry, and high-order aberration power were significantly reduced (30). The magnitude of astigmatism caused by pterygium depends on the traction force associated with corneal flattening caused by the horizontal extension length of the pterygium and its vascularity (31). Our study compared CD values 1 month after surgery with those before surgery and showed that pterygium excision not only changed the morphology of the cornea, but also significantly restored corneal transparency. The CD values of the anterior and full thickness of the cornea at 2–6, 6–10, and 0–12 mm, the center layer at 0–12 mm and the posterior layer at 0–2 mm after pterygium surgery in our study were significantly lower than those before surgery. Because pterygium mainly invades Descemet’s membrane and the superficial stromal layer of the cornea, it has a greater effect on CD of the anterior and center layer than posterior layer after pterygium excision. A previous study measured changes in corneal scar densities by Pentacam showed that CD significantly decreased in the anterior layer and increased in the posterior layer at 12 weeks compared with 1 week after pterygium surgery in 31 cases (32). And the CD values in the anterior layer continued to decrease 18 months after pterygium surgery (33). However, more postoperative time periods and more sample sizes are still needed to investigate the effect of pterygium surgery on CD at different layers of the cornea.

This study has some limitations. First, this study was retrospective, and few cases of mild pterygium were included. Although we analyzed the relationship between pterygium severity and CD value, more patients with mild pterygium are needed for further investigation. Second, as the pterygium tissue invades the cornea from the conjunctiva, the corneal optical density values analyzed in this study encompass 10–12 mm, however, this range may be affected by the corneoscleral limbus, and some patients have a corneal diameter below 12 mm, therefore, the reproducibility and predictability of this region are poor (13). Therefore, we analyzed the correlation between CD values and corneal parameters only at 0–2 and 2–6 mm from the cornea, which is more representative. Third, the follow-up period after the pterygium surgery was short. The difference was observed between one month after surgery and before surgery. Dynamic changes in CD values should be evaluated at multiple time points and over a longer follow-up period.

## Conclusion

This study demonstrated that CD values were higher in pterygium eyes than in the contralateral eyes, particularly in the anterior and central layers. CD values were correlated with pterygium severity and corneal parameters. Our findings show that pterygium excision combined with conjunctival autograft transplantation can reduce CD values. Dynamic long term postoperative changes in CD values and the effect of mild pterygium on CD values warrant further investigation.

## Data availability statement

The original contributions presented in this study are included in the article/supplementary material, further inquiries can be directed to the corresponding authors.

## Ethics statement

The studies involving human participants were reviewed and approved by Ethics Committee of Shenzhen Eye Hospital. The ethics committee waived the requirement of written informed consent for participation.

## Author contributions

JZ and LZ contributed to conception and design. JZ, LZ, HH, LS, and WH contributed to provision of study materials or patients, JZ, LZ, HH, LS, WH, ZZ, and JW contributed to collection and assembly of data. JZ, LZ, DN, and XL contributed to data analysis and interpretation. JZ, HH, and XL contributed to administrative support. All authors contributed to manuscript writing and final approval of manuscript.
